# Impact of prognostic nutritional index on long-term outcomes in patients with breast cancer

**DOI:** 10.1186/s12957-016-0920-7

**Published:** 2016-06-27

**Authors:** Tomomi Mohri, Yasuhiko Mohri, Tsunehiko Shigemori, Kenji Takeuchi, Yoshiyuki Itoh, Toshio Kato

**Affiliations:** Department of Surgery, Toyama Hospital, Tsu city, Mie 514-0043 Japan; Department of Gastrointestinal and Pediatric Surgery, Mie University Graduate School of Medicine, 2-174, Edobashi, Tsu city, Mie 514-0875 Japan

**Keywords:** Breast cancer, Prognostic nutritional index, Survival

## Abstract

**Background:**

Prognostic nutritional index has been shown to be a prognostic marker for various solid tumors. However, few studies have investigated the impact of the prognostic nutritional index on survival of patients with breast cancer. The aim of this study was to investigate the impact of the prognostic nutritional index on the long-term outcomes in patients with breast cancer.

**Methods:**

This study reviewed the medical records of 212 patients with breast cancer who underwent mastectomy. The prognostic nutritional index was calculated as 10 × serum albumin (g/dl) + 0.005 × total lymphocyte count (per mm^3^). Receiver operating characteristic curve analysis was performed to determine the cutoff value of the prognostic nutritional index. The survival curves were calculated by the Kaplan–Meier method. Differences between the curves were analyzed by the log-rank test. Multivariate Cox proportional hazard model was used to evaluate the prognostic significance of prognostic nutritional index in patients with breast cancer.

**Results:**

The mean prognostic nutritional index just before the operation was 51.9, and the median follow-up after surgery was 47.7 months. The optimal cutoff value of the prognostic nutritional index for predicting the overall survival was 52.8 from the receiver operating characteristic curve analysis. The 5-year overall survival rate was 98.3 % in the prognostic nutritional index >52.8 and 92.0 % in the prognostic nutritional index <52.8 (*P* = 0.013). In the multivariate analysis, a low prognostic nutritional index was an independent predictor for poor overall survival (HR, 5.88; 95 % CI, 1.13–108.01; *P* = 0.033).

**Conclusions:**

The prognostic nutritional index is a simple and useful marker for predicting the long-term outcomes of breast cancer patients, independent of the tumor stage.

**Electronic supplementary material:**

The online version of this article (doi:10.1186/s12957-016-0920-7) contains supplementary material, which is available to authorized users.

## Background

Breast cancer is the most frequently diagnosed malignancy and is the leading cause of cancer death among women [1]. The prognosis of breast cancer is influenced by well-recognized host- and tumor-related factors, including patient age, histological type and grade, tumor size, lymph node status, estrogen receptor (ER) and progesterone receptor (PR) status, and human epidermal growth factor receptor 2 (HER2) status [2]. Despite recent improvements in early detection, progress in surgical techniques, chemotherapy, and endocrine therapy, some patients with breast cancer develop recurrence, even after curative resection. Therefore, accurate prediction of prognosis is needed to improve patient survival and to provide important information to the patients.

Serum albumin is one of the most commonly used markers for assessing nutritional status. Albumin is produced by the liver and is the major protein in blood, acting as a key antioxidant, detoxifier, and transporter of important nutrients. In advanced cancer patients, the levels of serum albumin fall sharply because malnutrition and systematic inflammatory response to tumors both suppress albumin synthesis [3]. The prevalence of malnutrition among breast cancer patients reported by two studies was 20.5 % [4] and 18.3 % [5], respectively. A Korean study also showed that >51 % of female breast cancer patients had moderate to high risk of malnutrition [6]. Malnutrition can cause many clinical consequences, including decreased quality of life, reduced treatment response, and increased treatment-related toxicity. The prognostic nutritional index (PNI), which is based on serum albumin concentration and total peripheral lymphocyte count, was originally proposed to assess the perioperative immunological status and surgical risk in patients undergoing gastrointestinal surgery [7]. Recently, the PNI has been shown to be a prognostic marker for various solid tumors [8–10]. However, few studies have investigated the impact of the PNI on survival of patients with breast cancer. Therefore, we retrospectively investigated the correlation between the PNI and clinicopathological factors and the impact of the PNI on survival in breast cancer patients.

## Methods

### Patients

A total of 219 patients with histologically confirmed breast cancer underwent surgery between January 2006 and October 2015 at the Department of Surgery, Toyama Hospital, Japan. We excluded seven patients with distant metastasis. Therefore, 212 patients were analyzed in this study. The median age was 66 (range 27–96) years. Most patients received adjuvant chemotherapy and/or endocrine therapy according to the clinical practice guidelines for breast cancer from the Japanese Breast Cancer Society [11], if necessary.

### Data collection

Clinicopathological characteristics were obtained retrospectively from the medical records and evaluated as prognostic factors; these included patient age, tumor size, lymph node metastasis, pathological stage, hormonal receptor status (ER and PR), and HER2 status. The stage of breast cancer was classified according to the 7th edition of American Joint Committee on Cancer TNM Classification System [12].

We also collected data from blood tests just before the operation, including the level of serum albumin, total peripheral lymphocyte count, and carcinoembryonic antigen (CEA) and carbohydrate antigen (CA) 15-3 levels. PNI was calculated using the following formula: 10 × serum albumin value (g/dl) + 0.005 × total lymphocyte count in the peripheral blood (per mm^3^).

### Statistical analysis

The categorical variables were presented as numbers and percentages, and the groups were compared using the *χ*^2^ test. Continuous variables with normal distribution were expressed as the mean and standard deviation (SD) and were compared using the Mann–Whitney *U* test or Kruskal–Wallis test.

At the time of the final follow-up (February 2016), the median follow-up was 47.7 months. Overall survival (OS) was defined as the time from the operation until death. Disease-free survival (DFS) was defined as the time from the operation to disease recurrence. In DFS analysis, the patients who died of any other cause rather than breast cancer were excluded. The survival curves were calculated by the Kaplan–Meier method. Differences between the curves were analyzed by the log-rank test. To evaluate the sensitivity and specificity for OS and DFS, the receiver operating characteristic (ROC) curve was calculated, and the Youden index was estimated to determine the optimal cutoff value for the PNI. Univariate associations with long-term survival were determined using Kaplan–Meier analysis and the log-rank test. Multivariable analyses were performed by Cox proportional hazards regression analysis, incorporating all variables with *P* < 0.10 on univariate analysis. Statistical significance was defined as *P* < 0.05. These analyses were conducted using JMP version 11.0.0 (SAS Institute, Cary, NC, USA).

## Results

### PNI and clinicopathological characteristics of patients

The mean PNI just before the operation was 51.9 (SD 4.9). Most of the 212 patients had ductal carcinoma, 67 (32 %) had a tumor >2 cm, and 44 (21 %) had axillary lymph node metastasis. The mean PNI in patients aged >65 years was significantly lower than that in patients aged ≦65 years (*P* < 0.001; Table 1). The PNI value gradually decreased with the advancing disease stage, but significant difference was not observed (*P* = 0.124; Table 1).

**Table 1:** Relationship between clinicopathological factors and PNI

Variables		*n* (%)	PNI, mean ± SD	*P* value
Age (year)	≦65	102 (48)	53.1 ± 4.1	<0.001
	>65	110 (52)	50.8 ± 5.2	
Histology	Ductal	191 (90)	51.9 ± 4.7	0.291
	Lobular	9 (4)	49.2 ± 5.0	
	Special	12 (6)	53.4 ± 6.2	
Tumor size (cm)	≦2	145 (68)	52.3 ± 4.8	0.166
	>2, ≦5	61 (29)	50.9 ± 5.0	
	>5	6 (3)	51.7 ± 4.0	
Nodal metastasis	0	168 (79)	52.0 ± 5.0	0.240
	1–3	28 (13)	52.0 ± 4.4	
	>3	16 (8)	50.1 ± 3.7	
Stage	0/I	135 (64)	52.3 ± 4.8	0.124
	II	58 (27)	51.5 ± 4.9	
	III	19 (9)	50.5 ± 4.5	
Estrogen receptor status	Positive	168 (79)	51.8 ± 5.0	0.682
	Negative	44 (21)	52.3 ± 4.4	
Progesterone receptor status	Positive	145 (68)	51.8 ± 5.0	0.879
	Negative	67 (32)	52.1 ± 4.6	
HER2 status	Positive	39 (18)	51.0 ± 4.8	0.152
	Negative	173 (82)	52.1 ± 4.8	
CEA (ng/ml)	<5	164 (77)	51.9 ± 4.6	0.953
	≥5	48 (23)	51.9 ± 5.5	
CA 15-3 (U/ml)	<23	199 (94)	52.0 ± 4.8	0.327
	≥23	13 (6)	50.4 ± 5.1	

*PNI* prognostic nutritional index, *ER* estrogen receptor, *PR* progesterone receptor, *HER-2* human epidermal growth factor receptor 2

### ROC analysis

Using OS as an endpoint, the area under the ROC curve for the PNI was 0.676. When the PNI was 52.8, the Youden index was maximal, with a sensitivity of 91.7 % and specificity of 41.5 %. Therefore, the cutoff value of the PNI was set at 52.8. Then, 85 patients (40.0 %) with a PNI >52.8 and 127 patients (60.0 %) with a PNI ≤52.8 were classified into PNI-high and PNI-low groups, respectively. During the studied period, four patients died of any other cause rather than breast cancer. Those patients were excluded from DFS analysis. Using DFS as an endpoint, the area under the ROC curve for the PNI was 0.657. The cutoff value of the PNI on DFS was set at 52.4. There were 90 patients with a PNI >52.4 (a PNI-high group) and 118 patients with a PNI ≤52.4 (a PNI-low group).

### OS and DFS

**Fig. 1:**
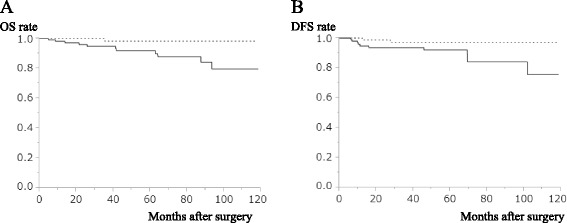
Kaplan–Meier estimates of OS and DFS according to the PNI. **a** OS rate of the PNI-low group was significantly lower than that of the PNI-high group (*P* = 0.019). **b** DFS rate of the PNI-low group was significantly lower than that of the PNI-high group (*P* = 0.035). *Solid line* indicates the PNI-low group, and *dotted line* indicates the PNI-high group

The 5-year OS rate was 98.3 % in the PNI-high group and 92.0 % in the PNI-low group (*P* = 0.019; Fig. 1a). One patient (1.2 %) died in the PNI-high group, and seven (5.5 %) died in the PNI-low group. The cause of death in the PNI-low group was tumor relapse in four patients, other cancer in two patients, and a cause other than cancer in one patient. One patient in PNI-high group died of a cause other than cancer. The 5-year DFS rate was 97.1 % in the PNI-high group and 92.0 % in the PNI-low group (*P* = 0.035; Fig. 1b).

### Predictive factors for OS

In the univariate analysis, tumor size, lymph node metastasis, and PNI were significantly associated with OS (Table 2). The multivariate analysis demonstrated that PNI and lymph node metastasis were independent prognostic factors for the OS (Table 3).

**Table 2:** Univariate analysis of prognostic factors for OS

Variables		5-year survival (%)	*P* value
Age (year)	≦65 >65	100 88.4	<0.001
Histological type	Ductal Lobular/Special	94.8 93.8	0.174
Tumor size (cm)	≤2 >2	95.9 91.7	0.005
Lymph node metastasis	Negative Positive	95.7 90.4	0.001
Estrogen receptor status	Negative Positive	94.3 94.8	0.827
Progesterone receptor status	Negative Positive	92.5 95.9	0.619
HER2 status	Negative Positive	95.3 90.8	0.967
CEA (ng/ml)	<5 ≥5	93.7 97.7	0.152
CA15-3 (U/ml)	<23 ≥23	94.9 88.9	0.101
PNI	>52.8 ≦52.8	98.3 92.0	0.019

*OS* overall survival, *ER* estrogen receptor, *PR* progesterone receptor, *HER-2* human epidermal growth factor receptor 2, *CEA* carcinoembryonic antigen, *CA 15-3* carbohydrate antigen 15-3, *PNI* prognostic nutritional index

**Table 3:** Cox proportional multivariate hazard models for OS

Variables		Hazard ratio	95 % CI	*P* value
Age	>65	11.9	2.23–220.81	0.002
Tumor size	>2 cm	1.94	0.45–8.91	0.377
Lymph node metastasis	Positive	4.06	1.05–17.86	0.042
PNI	≦52.8	5.88	1.13–108.01	0.033

*CI* confidence interval, *PNI* prognostic nutritional index

## Discussion

Our present study demonstrated that the PNI can predict the long-term outcomes of breast cancer patients, independent of the conventional TNM classification. The OS and DFS rates of the PNI-low group were significantly lower than those of the PNI-high group. The multivariate analysis performed in the present study demonstrated that the PNI was an independent predictor for the OS.

There are several known predictors of breast cancer prognosis, such as tumor size, histological type, lymph node involvement, hormonal receptor status, and HER2 status. However, it is of interest that there are other host-related factors, such as the PNI. Previous studies have reported an impact of the PNI on the long-term outcomes in several solid tumors, and various cutoff values for the PNI were used in those studies [8–10]. The cutoff value was usually set at 45, because PNI <45 is defined as moderate to severe malnutrition. However, the optimal cutoff value of the PNI to predict the long-term outcomes remains unclear. In the present study, we performed a ROC curve analysis, and the optimal cutoff value for the PNI was determined to be 52.8. When the PNI was 52.8, the sensitivity and specificity for the 5-year OS were 91.7 and 41.5 %, respectively. Few studies have determined the prognostic impact of the PNI in breast cancer patients. Only one study has shown that the preoperative PNI can be used as a simple and useful marker for predicting the long-term outcomes of triple-negative breast cancer [13]. The present study showed that a low preoperative PNI was associated with a higher risk of postoperative recurrence of breast cancer independent of hormonal receptor status and TNM stage.

The association between decreased PNI and poor survival of breast cancer is probably complex and largely unclear; however, possible explanations do exist. PNI is derived from the absolute albumin and absolute lymphocyte counts and is a routinely available laboratory test. One potential mechanism underlying the prognostic impact of PNI is that low PNI reflects hypoalbuminemia. Serum albumin has been used to assess disease severity, progression, and prognosis. Another factor is that lymphocytes play an important role in the host immune response to eradicate the formation and progression of tumors [14]. High lymphocytic infiltration is associated with improved survival, independent of clinicopathological characteristics, in primary operable ductal invasive breast cancer [15]. The importance of lymphocytes has been highlighted in several studies in which increasing infiltration of tumors with lymphocytes was associated with better response to cytotoxic treatment and prognosis in breast cancer patients [16–18]. Thus, low PNI may confer a survival advantage by tumor cells and lead to poorer outcome and increased recurrence.

The major limitations of the present study were the retrospective nature of the study and the single-center design. We were unable to exclude the possibility that unequal distribution of unidentified clinicopathological parameters in our patient cohort may have biased the results. Therefore, a large, prospective study should therefore be performed to confirm our findings.

## Conclusions

Our clinical observation shows that the PNI is associated with OS and DFS in patients with breast cancer. The PNI is an easy-to-determine, reproducible, and inexpensive test. It can be easily incorporated into routine use as a prognostic factor. Despite all these results, prospective studies evaluating PNI in a large series are required in this field.

## Abbreviations

CA, carbohydrate antigen; CEA, carcinoembryonic antigen; DFS, disease-free survival; ER, estrogen receptor; HER2, human epidermal growth factor receptor 2; OS, overall survival; PNI, prognostic nutritional index; PR, progesterone receptor; ROC, receiver operating characteristic; SD, standard deviation.

## Additional file

Additional file 1: The raw data of this study.
